# Poly[[triaqua­(μ_3_-pyridine-2,4,6-tri­car­boxyl­ato)gadolinium(III)] monohydrate]

**DOI:** 10.1107/S1600536809038793

**Published:** 2009-10-03

**Authors:** Hong-Sheng Wang, Wan-Qiang Zhang

**Affiliations:** a College of Chemistry and Chemical Engineering, Xuchang University, Xuchang, Henan Province 461000, People’s Republic of China

## Abstract

The title compound, {[Gd(C_8_H_2_NO_6_)(H_2_O)_3_]·H_2_O}_*n*_, was obtained in water under hydro­thermal conditions. The Gd^III^ ions are nine-coordinated by two O and one N atoms from one pyridine-2,4,6-tricarboxyl­ate ligand, two O atoms from another ligand, one O atom from a third ligand and three coordinated water mol­ecules. Each ligand binds three metal centers. Two-dimensional layers are formed through the Gd—O bonds and the layers are linked by O—H⋯O hydrogen bonds, forming a three-dimensional network.

## Related literature

For related structures, see: Gao *et al.* (2006 ▶); Ghosh & Bharadwaj (2005 ▶); Wang *et al.* (2007 ▶); Fu & Xu (2008 ▶); Li *et al.* (2008 ▶). For general background to lanthanide-organic frameworks and their properties, see: Parker (2000 ▶); Tobisch (2005 ▶); Pan *et al.* (2003 ▶). 
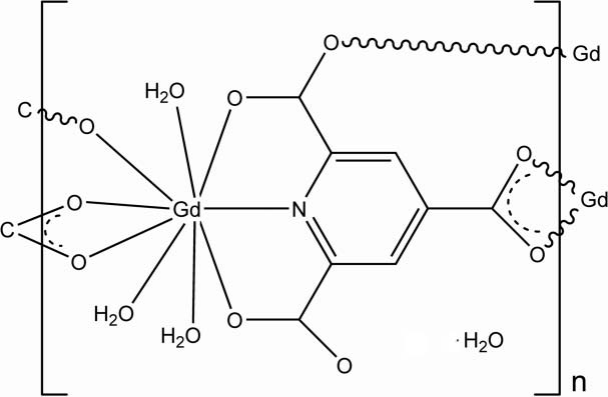


## Experimental

### 

#### Crystal data

[Gd(C_8_H_2_NO_6_)(H_2_O)_3_]·H_2_O
*M* *_r_* = 437.42
Monoclinic, 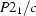

*a* = 11.896 (3) Å
*b* = 7.2696 (14) Å
*c* = 13.505 (3) Å
β = 96.259 (3)°
*V* = 1160.9 (4) Å^3^
*Z* = 4
Mo *K*α radiation
μ = 5.77 mm^−1^
*T* = 113 K
0.12 × 0.10 × 0.08 mm

#### Data collection

Rigaku Saturn diffractometer
Absorption correction: multi-scan (*REQAB*; Jacobson, 1998 ▶) *T*_min_ = 0.544, *T*_max_ = 0.655
10599 measured reflections
2776 independent reflections
2366 reflections with *I* > 2σ(*I*)
*R*_int_ = 0.038

#### Refinement

*R*[*F* ^2^ > 2σ(*F* ^2^)] = 0.021
*wR*(*F* ^2^) = 0.049
*S* = 1.04
2776 reflections
206 parameters
8 restraints
H atoms treated by a mixture of independent and constrained refinement
Δρ_max_ = 0.64 e Å^−3^
Δρ_min_ = −1.29 e Å^−3^

### 

Data collection: *CrystalClear* (Rigaku/MSC, 2005 ▶); cell refinement: *CrystalClear*; data reduction: *CrystalClear*; program(s) used to solve structure: *SHELXS97* (Sheldrick, 2008 ▶); program(s) used to refine structure: *SHELXL97* (Sheldrick, 2008 ▶); molecular graphics: *SHELXTL* (Sheldrick, 2008 ▶); software used to prepare material for publication: *CrystalStructure* (Rigaku/MSC, 2005 ▶).

## Supplementary Material

Crystal structure: contains datablocks I, global. DOI: 10.1107/S1600536809038793/zq2008sup1.cif

Structure factors: contains datablocks I. DOI: 10.1107/S1600536809038793/zq2008Isup2.hkl

Additional supplementary materials:  crystallographic information; 3D view; checkCIF report

## Figures and Tables

**Table 1:** Hydrogen-bond geometry (Å, °)

*D*—H⋯*A*	*D*—H	H⋯*A*	*D*⋯*A*	*D*—H⋯*A*
O7—H7*A*⋯O1^i^	0.82 (2)	2.02 (3)	2.794 (3)	157 (4)
O7—H7*B*⋯O3^ii^	0.83 (2)	1.97 (2)	2.795 (3)	175 (4)
O8—H8*A*⋯O6^iii^	0.82 (3)	1.80 (3)	2.621 (3)	171 (4)
O8—H8*B*⋯O4^iv^	0.75 (2)	2.22 (3)	2.933 (3)	158 (4)
O9—H9*A*⋯O6^iii^	0.82 (2)	2.01 (3)	2.800 (3)	161 (4)
O9—H9*B*⋯O10	0.83 (2)	1.91 (3)	2.723 (3)	166 (4)
O10—H10*A*⋯O8^iv^	0.81 (2)	2.24 (3)	3.051 (4)	173 (4)
O10—H10*B*⋯O9^v^	0.82 (3)	2.45 (3)	3.169 (4)	148 (4)

Symmetry codes: (i) 
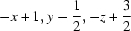
; (ii) 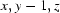; (iii) 
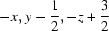
; (iv) 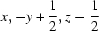; (v) 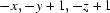.

**Fig. 1.**
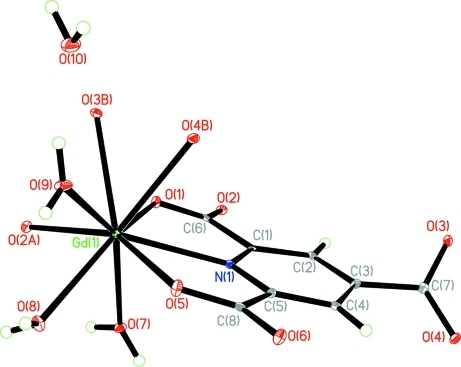
The molecular structure of (I), with atom labels and 30% probability displacement ellipsoids for non-H atoms.

**Fig. 2.**
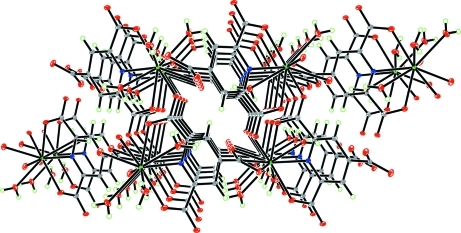
The packing of (I), showing the two-dimensional layers formed by Gd—O bonds.

**Fig. 3.**
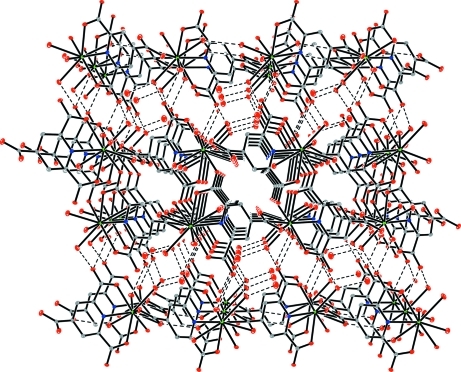
View of the three-dimensional network constructed by O—H···O hydrogen bonds (dashed lines). All H atoms were omitted for clarity.

## supplementary crystallographic information

### Comment

The preparation and property researching of metal-organic frameworks have attracted widespread interest in recent years due to their potential application in the areas of magnetism, luminescence, adsorption, catalysis and so on (Parker, 2000; Tobisch, 2005; Pan *et al.*, 2003). Multicarboxylic acids containing pyridyl rings were widely used and many 1-D, 2-D and 3-D coordination polymers with novel structures have been reported. Especially, complexes with pyridine-2,4,6-tricarboxylato (H_3_pta = pyridine-2,4,6-tricarboxylic acid) ligands have been recently reported (Li *et al.*, 2008; Wang *et al.*, 2007; Fu *et al.*, 2008.). The title compound is a new Gd^III^ complex built with pta ligands and prepared under hydrothermal conditions.

As shown in Fig. 1, the local geometry of Gd^III^ ion is a distorted monocapped antitetragonal prism. Each pta ligand connects three Gd^III^ ions with oxgen atoms of the carboxyl groups and the nitrogen atom. There are three coordination water molecules on each Gd^III^ ion. A two-dimentional layer is constructed by the bonding among oxygen atoms and Gd^III^ ions (see Fig. 2). In addition, a lattice water molecule per asymmetric unit is in the crystal structure. Many O—H···O hydrogen bonds are formed between the oxygen atoms of water molecules and the oxygen atoms of caboxyl groups. As a result, the three-dimensional network formed by hydrogen bonds is shown in Fig. 3.

### Experimental

A mixture of H_3_pta (0.0422 g, 0.2 mmol), GdCl_3_.6H_2_O (0.0743 g, 0.2 mmol) and deionized water (15 ml) was put in a teflon-lined steel bomb and heated at 453 K for 3 days, then cooled the bomb at a rate of 2 K/hour. The colorless crystals suitable for X-ray diffraction measurements were obtained. Spectroscopic analysis: IR (KBr, ν cm^-1^): 3606, 3382, 1631, 1608, 1582, 1549, 1445, 1395, 1352, 1277, 1235, 1110, 1025, 950, 931, 818, 791, 740, 664, 623, 587, 543, 479, 435. Elemental analysis, calculated for C_8_H_10_GdNO_10_: C, 21.97; H, 2.30; N, 3.20.%; found: C, 22.18; H, 2.11; N, 3.54%.

### Refinement

All hydrogen atoms bonded to carbon atoms were positioned geometrically and refined as riding, with C—H = 0.95 Å and *U*_iso_(H) = 1.2*U*_eq_(C). The H atoms of water molecules were found from difference Fourier maps and included in the final refinements with a restraint of O—H = 0.75 - 0.85 Å and *U*_iso_(H) = 1.5 *U*_eq_(O).

### Crystal data

**Table:**

[Gd(C_8_H_2_NO_6_)(H_2_O)_3_]·H_2_O	*F*(000) = 836
*M**_r_* = 437.42	*D*_x_ = 2.503 Mg m^−^^3^
Monoclinic, *P*2_1_/*c*	Mo *K*α radiation, λ = 0.71070 Å
Hall symbol: -P 2ybc	Cell parameters from 3775 reflections
*a* = 11.896 (3) Å	θ = 1.7–28.7°
*b* = 7.2696 (14) Å	µ = 5.77 mm^−^^1^
*c* = 13.505 (3) Å	*T* = 113 K
β = 96.259 (3)°	Block, colourless
*V* = 1160.9 (4) Å^3^	0.12 × 0.10 × 0.08 mm
*Z* = 4	

### Data collection

**Table:**

Rigaku Saturn diffractometer	2776 independent reflections
Radiation source: rotating anode	2366 reflections with *I* > 2σ(*I*)
confocal	*R*_int_ = 0.038
Detector resolution: 7.31 pixels mm^-1^	θ_max_ = 27.9°, θ_min_ = 1.7°
ω scans	*h* = −15→15
Absorption correction: multi-scan (REQAB; Jacobson, 1998)	*k* = −8→9
*T*_min_ = 0.544, *T*_max_ = 0.655	*l* = −17→17
10599 measured reflections	

### Refinement

**Table:**

Refinement on *F*^2^	Primary atom site location: structure-invariant direct methods
Least-squares matrix: full	Secondary atom site location: difference Fourier map
*R*[*F*^2^ > 2σ(*F*^2^)] = 0.021	Hydrogen site location: inferred from neighbouring sites
*wR*(*F*^2^) = 0.049	H atoms treated by a mixture of independent and constrained refinement
*S* = 1.04	*w* = 1/[σ^2^(*F*_o_^2^) + (0.0242*P*)^2^] where *P* = (*F*_o_^2^ + 2*F*_c_^2^)/3
2776 reflections	(Δ/σ)_max_ = 0.001
206 parameters	Δρ_max_ = 0.64 e Å^−^^3^
8 restraints	Δρ_min_ = −1.29 e Å^−^^3^

### Special details

**Table:**

Geometry. All e.s.d.'s (except the e.s.d. in the dihedral angle between two l.s. planes) are estimated using the full covariance matrix. The cell e.s.d.'s are taken into account individually in the estimation of e.s.d.'s in distances, angles and torsion angles; correlations between e.s.d.'s in cell parameters are only used when they are defined by crystal symmetry. An approximate (isotropic) treatment of cell e.s.d.'s is used for estimating e.s.d.'s involving l.s. planes.
Refinement. Refinement of *F*^2^ against ALL reflections. The weighted *R*-factor *wR* and goodness of fit *S* are based on *F*^2^, conventional *R*-factors *R* are based on *F*, with *F* set to zero for negative *F*^2^. The threshold expression of *F*^2^ > σ(*F*^2^) is used only for calculating *R*-factors(gt) *etc*. and is not relevant to the choice of reflections for refinement. *R*-factors based on *F*^2^ are statistically about twice as large as those based on *F*, and *R*- factors based on ALL data will be even larger.

### Fractional atomic coordinates and isotropic or equivalent isotropic displacement parameters (Å^2^)

**Table:**

	*x*	*y*	*z*	*U*_iso_*/*U*_eq_	
Gd1	0.283337 (12)	0.28362 (2)	0.703522 (11)	0.00496 (6)	
O1	0.46782 (18)	0.4287 (3)	0.74763 (16)	0.0085 (5)	
O2	0.58731 (18)	0.5825 (3)	0.85710 (16)	0.0080 (4)	
O3	0.3449 (2)	1.0594 (3)	1.05119 (16)	0.0101 (5)	
O4	0.25022 (19)	0.8881 (3)	1.14877 (16)	0.0111 (5)	
O5	0.10951 (19)	0.3347 (3)	0.77338 (17)	0.0118 (5)	
O6	0.00718 (19)	0.4715 (3)	0.88248 (16)	0.0105 (5)	
O7	0.3762 (2)	0.1238 (3)	0.85200 (17)	0.0109 (5)	
H7A	0.425 (3)	0.051 (4)	0.838 (3)	0.016*	
H7B	0.369 (3)	0.110 (5)	0.9117 (19)	0.016*	
O8	0.1937 (2)	−0.0211 (3)	0.71872 (18)	0.0117 (5)	
H8A	0.129 (2)	−0.034 (5)	0.690 (3)	0.018*	
H8B	0.225 (3)	−0.109 (4)	0.709 (3)	0.018*	
O9	0.1329 (2)	0.2581 (3)	0.56658 (18)	0.0135 (5)	
H9A	0.081 (3)	0.185 (4)	0.571 (3)	0.020*	
H9B	0.121 (3)	0.314 (5)	0.513 (2)	0.020*	
O10	0.0781 (2)	0.4862 (3)	0.40873 (19)	0.0194 (6)	
H10A	0.106 (4)	0.505 (6)	0.357 (2)	0.029*	
H10B	0.016 (3)	0.531 (5)	0.392 (3)	0.029*	
N1	0.2963 (2)	0.4907 (3)	0.85098 (19)	0.0060 (5)	
C1	0.3938 (3)	0.5761 (4)	0.8822 (2)	0.0055 (6)	
C2	0.4007 (3)	0.7107 (4)	0.9553 (2)	0.0073 (6)	
H2	0.4699	0.7730	0.9744	0.009*	
C3	0.3035 (3)	0.7525 (4)	1.0003 (2)	0.0082 (6)	
C4	0.2043 (3)	0.6563 (4)	0.9712 (2)	0.0073 (6)	
H4	0.1385	0.6761	1.0037	0.009*	
C5	0.2035 (3)	0.5320 (4)	0.8944 (2)	0.0070 (6)	
C6	0.4912 (3)	0.5242 (4)	0.8255 (2)	0.0068 (6)	
C7	0.3007 (3)	0.9081 (4)	1.0720 (2)	0.0077 (6)	
C8	0.0974 (3)	0.4387 (4)	0.8469 (2)	0.0072 (6)	

### Atomic displacement parameters (Å^2^)

**Table:**

	*U*^11^	*U*^22^	*U*^33^	*U*^12^	*U*^13^	*U*^23^
Gd1	0.00489 (8)	0.00531 (9)	0.00486 (9)	0.00011 (6)	0.00130 (6)	−0.00021 (6)
O1	0.0066 (11)	0.0089 (11)	0.0103 (12)	−0.0013 (8)	0.0017 (9)	−0.0024 (9)
O2	0.0051 (11)	0.0103 (11)	0.0082 (11)	−0.0021 (8)	−0.0006 (9)	0.0004 (8)
O3	0.0137 (12)	0.0071 (11)	0.0099 (12)	−0.0012 (9)	0.0034 (9)	−0.0026 (9)
O4	0.0143 (13)	0.0104 (12)	0.0094 (12)	−0.0038 (9)	0.0059 (9)	−0.0025 (9)
O5	0.0075 (12)	0.0142 (11)	0.0142 (12)	−0.0027 (9)	0.0030 (9)	−0.0062 (9)
O6	0.0054 (11)	0.0150 (12)	0.0111 (12)	0.0014 (9)	0.0015 (9)	−0.0024 (9)
O7	0.0132 (13)	0.0111 (12)	0.0086 (12)	0.0047 (9)	0.0023 (10)	0.0035 (9)
O8	0.0084 (12)	0.0096 (12)	0.0166 (13)	0.0008 (9)	−0.0005 (10)	−0.0012 (10)
O9	0.0135 (13)	0.0173 (13)	0.0088 (12)	−0.0061 (9)	−0.0027 (10)	0.0041 (9)
O10	0.0237 (16)	0.0199 (13)	0.0141 (14)	0.0057 (11)	0.0010 (12)	0.0048 (11)
N1	0.0057 (13)	0.0058 (12)	0.0065 (13)	0.0006 (9)	0.0012 (10)	0.0008 (10)
C1	0.0072 (15)	0.0040 (14)	0.0056 (15)	−0.0021 (11)	0.0024 (12)	0.0023 (11)
C2	0.0071 (15)	0.0054 (14)	0.0094 (16)	−0.0029 (11)	0.0015 (12)	0.0004 (12)
C3	0.0097 (17)	0.0102 (16)	0.0042 (15)	0.0004 (11)	−0.0011 (12)	0.0011 (11)
C4	0.0061 (15)	0.0070 (14)	0.0092 (16)	0.0013 (11)	0.0026 (12)	0.0021 (12)
C5	0.0059 (15)	0.0073 (15)	0.0083 (16)	0.0010 (11)	0.0021 (12)	0.0009 (12)
C6	0.0095 (16)	0.0038 (14)	0.0071 (16)	0.0000 (11)	0.0007 (12)	0.0014 (11)
C7	0.0054 (15)	0.0101 (15)	0.0070 (16)	0.0018 (11)	−0.0017 (12)	0.0000 (12)
C8	0.0065 (16)	0.0060 (15)	0.0095 (16)	−0.0008 (11)	0.0023 (12)	0.0021 (12)

### Geometric parameters (Å, °)

**Table:**

Gd1—O2^i^	2.335 (2)	O7—H7B	0.83 (2)
Gd1—O5	2.393 (2)	O8—H8A	0.82 (3)
Gd1—O9	2.437 (3)	O8—H8B	0.75 (2)
Gd1—O1	2.449 (2)	O9—H9A	0.82 (2)
Gd1—O7	2.471 (2)	O9—H9B	0.83 (2)
Gd1—O8	2.477 (2)	O10—H10A	0.81 (2)
Gd1—N1	2.488 (2)	O10—H10B	0.82 (3)
Gd1—O4^ii^	2.517 (2)	N1—C5	1.339 (4)
Gd1—O3^ii^	2.530 (2)	N1—C1	1.342 (4)
Gd1—C7^ii^	2.880 (3)	C1—C2	1.386 (4)
O1—C6	1.266 (4)	C1—C6	1.504 (4)
O2—C6	1.250 (4)	C2—C3	1.397 (4)
O2—Gd1^iii^	2.335 (2)	C2—H2	0.9500
O3—C7	1.264 (4)	C3—C4	1.391 (4)
O3—Gd1^iv^	2.530 (2)	C3—C7	1.492 (4)
O4—C7	1.261 (4)	C4—C5	1.374 (4)
O4—Gd1^iv^	2.517 (2)	C4—H4	0.9500
O5—C8	1.269 (4)	C5—C8	1.513 (4)
O6—C8	1.245 (4)	C7—Gd1^iv^	2.880 (3)
O7—H7A	0.82 (2)		
			
O2^i^—Gd1—O5	150.00 (8)	C6—O1—Gd1	123.22 (19)
O2^i^—Gd1—O9	98.27 (8)	C6—O2—Gd1^iii^	134.9 (2)
O5—Gd1—O9	73.52 (8)	C7—O3—Gd1^iv^	92.69 (17)
O2^i^—Gd1—O1	75.39 (7)	C7—O4—Gd1^iv^	93.35 (18)
O5—Gd1—O1	128.84 (7)	C8—O5—Gd1	125.3 (2)
O9—Gd1—O1	141.45 (7)	Gd1—O7—H7A	112 (3)
O2^i^—Gd1—O7	74.73 (7)	Gd1—O7—H7B	140 (3)
O5—Gd1—O7	94.71 (8)	H7A—O7—H7B	107 (4)
O9—Gd1—O7	143.75 (8)	Gd1—O8—H8A	117 (3)
O1—Gd1—O7	72.28 (7)	Gd1—O8—H8B	121 (3)
O2^i^—Gd1—O8	77.00 (7)	H8A—O8—H8B	106 (4)
O5—Gd1—O8	73.01 (8)	Gd1—O9—H9A	119 (3)
O9—Gd1—O8	72.98 (8)	Gd1—O9—H9B	131 (3)
O1—Gd1—O8	138.37 (7)	H9A—O9—H9B	109 (4)
O7—Gd1—O8	70.78 (8)	H10A—O10—H10B	98 (4)
O2^i^—Gd1—N1	132.39 (8)	C5—N1—C1	118.9 (3)
O5—Gd1—N1	64.62 (8)	C5—N1—Gd1	120.3 (2)
O9—Gd1—N1	129.04 (8)	C1—N1—Gd1	120.47 (19)
O1—Gd1—N1	64.48 (7)	N1—C1—C2	122.2 (3)
O7—Gd1—N1	69.63 (8)	N1—C1—C6	114.3 (3)
O8—Gd1—N1	117.68 (8)	C2—C1—C6	123.4 (3)
O2^i^—Gd1—O4^ii^	125.38 (7)	C1—C2—C3	118.4 (3)
O5—Gd1—O4^ii^	81.63 (7)	C1—C2—H2	120.8
O9—Gd1—O4^ii^	76.75 (8)	C3—C2—H2	120.8
O1—Gd1—O4^ii^	76.74 (7)	C4—C3—C2	119.0 (3)
O7—Gd1—O4^ii^	136.55 (8)	C4—C3—C7	119.1 (3)
O8—Gd1—O4^ii^	144.83 (8)	C2—C3—C7	121.7 (3)
N1—Gd1—O4^ii^	69.84 (8)	C5—C4—C3	118.7 (3)
O2^i^—Gd1—O3^ii^	74.76 (7)	C5—C4—H4	120.7
O5—Gd1—O3^ii^	126.13 (8)	C3—C4—H4	120.7
O9—Gd1—O3^ii^	70.79 (8)	N1—C5—C4	122.7 (3)
O1—Gd1—O3^ii^	70.88 (7)	N1—C5—C8	113.8 (3)
O7—Gd1—O3^ii^	136.76 (8)	C4—C5—C8	123.4 (3)
O8—Gd1—O3^ii^	129.52 (7)	O2—C6—O1	125.4 (3)
N1—Gd1—O3^ii^	112.32 (8)	O2—C6—C1	117.9 (3)
O4^ii^—Gd1—O3^ii^	51.91 (7)	O1—C6—C1	116.7 (3)
O2^i^—Gd1—C7^ii^	100.19 (8)	O4—C7—O3	122.0 (3)
O5—Gd1—C7^ii^	104.20 (8)	O4—C7—C3	119.5 (3)
O9—Gd1—C7^ii^	71.79 (8)	O3—C7—C3	118.4 (3)
O1—Gd1—C7^ii^	72.09 (8)	O4—C7—Gd1^iv^	60.73 (15)
O7—Gd1—C7^ii^	144.11 (8)	O3—C7—Gd1^iv^	61.32 (15)
O8—Gd1—C7^ii^	143.83 (9)	C3—C7—Gd1^iv^	176.6 (2)
N1—Gd1—C7^ii^	91.16 (8)	O6—C8—O5	126.3 (3)
O4^ii^—Gd1—C7^ii^	25.92 (7)	O6—C8—C5	117.7 (3)
O3^ii^—Gd1—C7^ii^	25.99 (7)	O5—C8—C5	116.0 (3)
			
O2^i^—Gd1—O1—C6	−147.2 (2)	Gd1—N1—C1—C2	171.2 (2)
O5—Gd1—O1—C6	12.7 (3)	C5—N1—C1—C6	−177.8 (3)
O9—Gd1—O1—C6	127.9 (2)	Gd1—N1—C1—C6	−4.0 (3)
O7—Gd1—O1—C6	−68.8 (2)	N1—C1—C2—C3	2.6 (4)
O8—Gd1—O1—C6	−97.2 (2)	C6—C1—C2—C3	177.5 (3)
N1—Gd1—O1—C6	6.5 (2)	C1—C2—C3—C4	0.9 (4)
O4^ii^—Gd1—O1—C6	80.3 (2)	C1—C2—C3—C7	−173.7 (3)
O3^ii^—Gd1—O1—C6	134.2 (2)	C2—C3—C4—C5	−4.3 (5)
C7^ii^—Gd1—O1—C6	106.8 (2)	C7—C3—C4—C5	170.5 (3)
O2^i^—Gd1—O5—C8	133.9 (2)	C1—N1—C5—C4	−1.1 (4)
O9—Gd1—O5—C8	−148.4 (3)	Gd1—N1—C5—C4	−174.9 (2)
O1—Gd1—O5—C8	−4.4 (3)	C1—N1—C5—C8	175.1 (3)
O7—Gd1—O5—C8	66.6 (2)	Gd1—N1—C5—C8	1.3 (3)
O8—Gd1—O5—C8	134.8 (3)	C3—C4—C5—N1	4.6 (5)
N1—Gd1—O5—C8	1.8 (2)	C3—C4—C5—C8	−171.3 (3)
O4^ii^—Gd1—O5—C8	−69.8 (2)	Gd1^iii^—O2—C6—O1	62.9 (4)
O3^ii^—Gd1—O5—C8	−98.3 (2)	Gd1^iii^—O2—C6—C1	−115.0 (3)
C7^ii^—Gd1—O5—C8	−82.7 (2)	Gd1—O1—C6—O2	171.1 (2)
O2^i^—Gd1—N1—C5	−151.4 (2)	Gd1—O1—C6—C1	−11.0 (3)
O5—Gd1—N1—C5	−1.5 (2)	N1—C1—C6—O2	−172.4 (3)
O9—Gd1—N1—C5	36.4 (3)	C2—C1—C6—O2	12.4 (4)
O1—Gd1—N1—C5	173.1 (2)	N1—C1—C6—O1	9.6 (4)
O7—Gd1—N1—C5	−107.4 (2)	C2—C1—C6—O1	−165.6 (3)
O8—Gd1—N1—C5	−53.6 (2)	Gd1^iv^—O4—C7—O3	0.5 (3)
O4^ii^—Gd1—N1—C5	88.6 (2)	Gd1^iv^—O4—C7—C3	−176.1 (3)
O3^ii^—Gd1—N1—C5	119.2 (2)	Gd1^iv^—O3—C7—O4	−0.5 (3)
C7^ii^—Gd1—N1—C5	103.6 (2)	Gd1^iv^—O3—C7—C3	176.1 (3)
O2^i^—Gd1—N1—C1	34.9 (3)	C4—C3—C7—O4	45.8 (4)
O5—Gd1—N1—C1	−175.3 (2)	C2—C3—C7—O4	−139.6 (3)
O9—Gd1—N1—C1	−137.4 (2)	C4—C3—C7—O3	−131.0 (3)
O1—Gd1—N1—C1	−0.6 (2)	C2—C3—C7—O3	43.6 (4)
O7—Gd1—N1—C1	78.9 (2)	Gd1—O5—C8—O6	177.7 (2)
O8—Gd1—N1—C1	132.6 (2)	Gd1—O5—C8—C5	−1.8 (4)
O4^ii^—Gd1—N1—C1	−85.1 (2)	N1—C5—C8—O6	−179.3 (3)
O3^ii^—Gd1—N1—C1	−54.5 (2)	C4—C5—C8—O6	−3.1 (4)
C7^ii^—Gd1—N1—C1	−70.1 (2)	N1—C5—C8—O5	0.2 (4)
C5—N1—C1—C2	−2.6 (4)	C4—C5—C8—O5	176.4 (3)

Symmetry codes: (i) −*x*+1, *y*−1/2, −*z*+3/2; (ii) *x*, −*y*+3/2, *z*−1/2; (iii) −*x*+1, *y*+1/2, −*z*+3/2; (iv) *x*, −*y*+3/2, *z*+1/2.

### Hydrogen-bond geometry (Å, °)

**Table:**

*D*—H···*A*	*D*—H	H···*A*	*D*···*A*	*D*—H···*A*
O7—H7A···O1^i^	0.82 (2)	2.02 (3)	2.794 (3)	157 (4)
O7—H7B···O3^v^	0.83 (2)	1.97 (2)	2.795 (3)	175 (4)
O8—H8A···O6^vi^	0.82 (3)	1.80 (3)	2.621 (3)	171 (4)
O8—H8B···O4^vii^	0.75 (2)	2.22 (3)	2.933 (3)	158 (4)
O9—H9A···O6^vi^	0.82 (2)	2.01 (3)	2.800 (3)	161 (4)
O9—H9B···O10	0.83 (2)	1.91 (3)	2.723 (3)	166 (4)
O10—H10A···O8^vii^	0.81 (2)	2.24 (3)	3.051 (4)	173 (4)
O10—H10B···O9^viii^	0.82 (3)	2.45 (3)	3.169 (4)	148 (4)

Symmetry codes: (i) −*x*+1, *y*−1/2, −*z*+3/2; (v) *x*, *y*−1, *z*; (vi) −*x*, *y*−1/2, −*z*+3/2; (vii) *x*, −*y*+1/2, *z*−1/2; (viii) −*x*, −*y*+1, −*z*+1.
